# Calculation of Similarity Between 26 Autoimmune Diseases Based on Three Measurements Including Network, Function, and Semantics

**DOI:** 10.3389/fgene.2021.758041

**Published:** 2021-11-11

**Authors:** Yanjun Ding, Mintian Cui, Jun Qian, Chao Wang, Qi Shen, Hongbiao Ren, Liangshuang Li, Fengmin Zhang, Ruijie Zhang

**Affiliations:** ^1^ College of Bioinformatics Science and Technology, Harbin Medical University, Harbin, China; ^2^ Department of Microbiology, WU Lien-Teh Institute, Harbin Medical University, Harbin, China

**Keywords:** ADs, genetic susceptibility, network similarity, functional similarity, semantic similarity, autoimmune tautology

## Abstract

Autoimmune diseases (ADs) are a broad range of diseases in which the immune response to self-antigens causes damage or disorder of tissues, and the genetic susceptibility is regarded as the key etiology of ADs. Accumulating evidence has suggested that there are certain commonalities among different ADs. However, the theoretical research about similarity between ADs is still limited. In this work, we first computed the genetic similarity between 26 ADs based on three measurements: network similarity (NetSim), functional similarity (FunSim), and semantic similarity (SemSim), and systematically identified three significant pairs of similar ADs: rheumatoid arthritis (RA) and systemic lupus erythematosus (SLE), myasthenia gravis (MG) and autoimmune thyroiditis (AIT), and autoimmune polyendocrinopathies (AP) and uveomeningoencephalitic syndrome (Vogt-Koyanagi-Harada syndrome, VKH). Then we investigated the gene ontology terms and pathways enriched by the three significant AD pairs through functional analysis. By the cluster analysis on the similarity matrix of 26 ADs, we embedded the three significant AD pairs in three different disease clusters respectively, and the ADs of each disease cluster might have high genetic similarity. We also detected the risk genes in common among the ADs which belonged to the same disease cluster. Overall, our findings will provide significant insight in the commonalities of different ADs in genetics, and contribute to the discovery of novel biomarkers and the development of new therapeutic methods for ADs.

## Introduction

Autoimmune diseases (ADs) are characterized by causing abnormal immune response which can damage human tissues as a result of the loss of immune tolerance to self-antigens (Margo and Harman, 2016). ADs affect more than 5% of the global population, the incidence and mortality of which have also increased markedly (Ji et al., 2016). Possible causes contain genetic, environmental, hormonal, and immunological factors (Stojanovich and Marisavljevich, 2008). However, neither the inner mechanism action nor the etiology of ADs is clear and there is still no effective cure for these diseases (Rosenblum et al., 2012; Li et al., 2017).

ADs share several clinical signs and symptoms, physiopathological mechanisms, and environmental and genetic factors, and this fact indicates that they have a common origin, which has been called the autoimmune tautology. A growing body of evidence has indicated the existence of the autoimmune tautology among various ADs: 1) Different ADs exhibit the same phenotypic characteristics (Anaya, 2017). These diseases, whether organ-specific or systematic, show tissue and organ damage and inflammatory pathological features (Place and Kanneganti, 2020). 2) Different ADs exhibit the same clinical characteristics. Clinically, the results from serological examinations of patients often overlap. And the same patient may suffer from two or more ADs simultaneously, which has been called the polyautoimmunity (PolyA) (Anaya, 2014). In addition, there is a tendency for ADs to cluster within families (Cardenas-Roldan et al., 2013). 3) Different ADs exhibit the same genetic characteristics. ADs are caused by the mutation of multiple loci in the human genome and share the same main genetic loci (Anaya et al., 2006). For example, a previous study indicated that the human leukocyte antigen (HLA) is a susceptibility gene shared by multiple ADs (Cruz-Tapias et al., 2012). And Ueda et al. (2003) found that a molecule encoded by *CTLA4* was vital for negative regulation of the immune system and could enhance the risk of several ADs, such as Graves disease, autoimmune hypothyroidism, and type 1 diabetes mellitus (T1D), which indicated that ADs might share similar pathogenic mechanisms. Li et al. (2015) proved that different pediatric ADs shared the same genetic variation. They analyzed the clinical cases of ten different ADs and found many of these diseases were familial and the patients often suffered from several ADs at the same time. In this study, 27 significant risk genetic loci were identified, of which 22 were shared by at least two ADs and 19 loci were shared by at least three ADs. Thus, identification of risk genes shared by multiple ADs may help to explain the development of PolyA. 4) In addition, different ADs also exhibit the same epigenetic characteristics. Epigenetic researches found that ADs shared similar epigenetic mechanisms. For instance, the DNA promoter region in the target cells of systemic lupus erythematosus (SLE) and rheumatoid arthritis (RA) showed low methylation (Quintero-Ronderos and Montoya-Ortiz, 2012). The strong similarity among ADs provides us with a deeper understanding of the common underlying mechanisms of ADs, and also prompts researchers to classify ADs. Therefore, the studies on the genetic similarity of ADs can help us to dissect AD pathogenesis, and contribute to the discovery of novel biomarkers and the development of new therapeutic methods for ADs, which is extremely important in clinical research.

**FIGURE 1:**
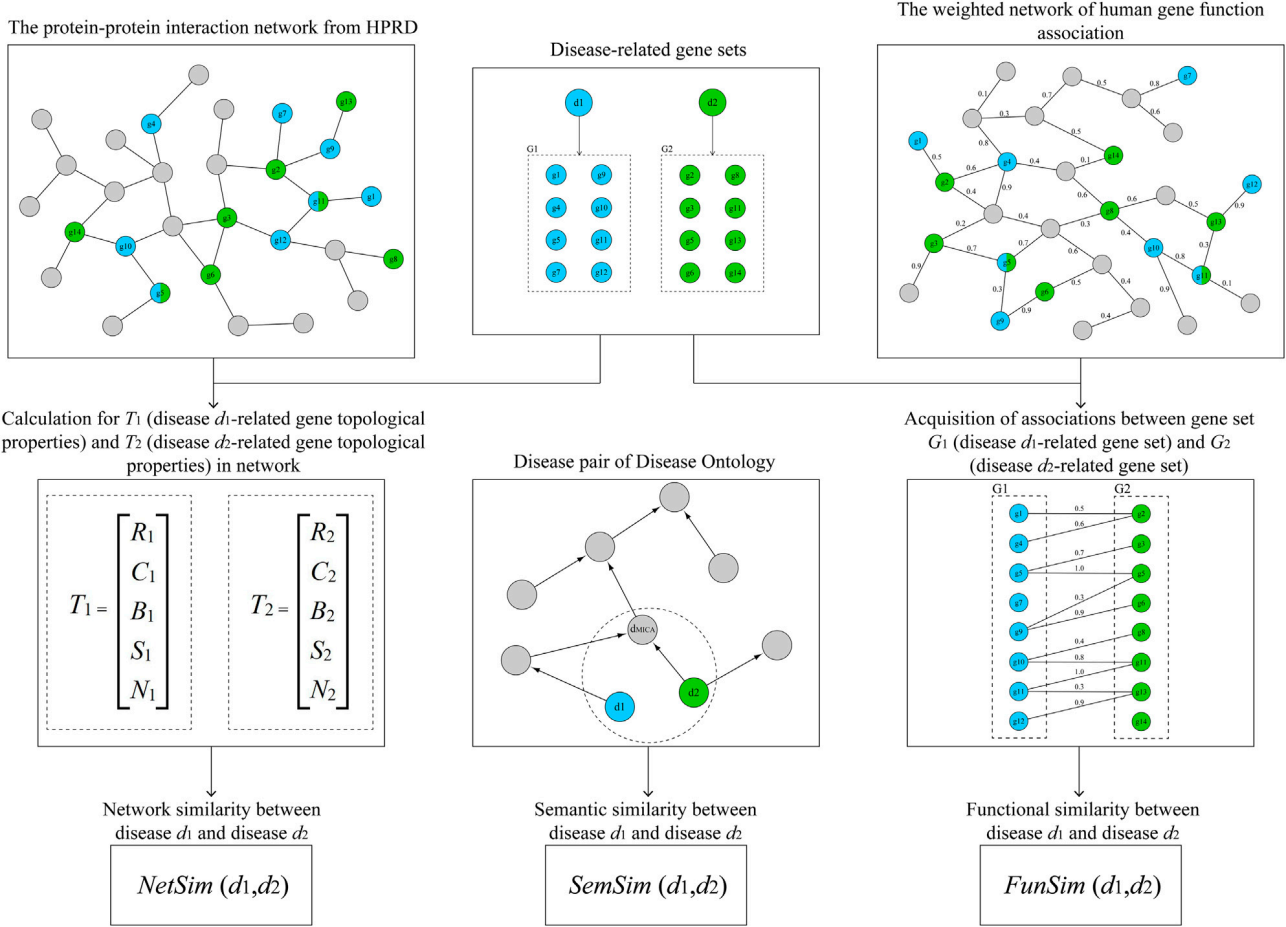
The workflow diagram for this study.

In this study, we utilized three measurements, including network similarity, functional similarity, and semantic similarity, to analyze genetic similarity between 26 ADs (the workflow diagram is shown in Figure 1). We identified three significant pairs of similar ADs by multi-step computational approaches. Besides, based on the similarity matrix of 26 ADs, we found some other ADs which were similar to significant pairs of similar ADs by cluster analysis. And the risk genes shared by the ADs which belonged to the same disease cluster could be promising biomarkers for ADs. Our findings provided a novel perspective to understand the commonalities of different ADs in genetics and would facilitate AD mechanism research.

## Materials and Methods

### Collection of AD Terms and AD-Related Genes

The AD terms (category C20.111), including 68 diseases, were acquired from the Medical Subject Headings (MeSH, https://www.nlm.nih.gov/mesh/meshhome.html). After removing the complications of ADs, we extracted human disease-related genes from the Genetic Association Database (GAD, https://geneticassociationdb.nih.gov/) (Becker et al., 2004) and mapped these genes to the AD terms for integration. The disease gene sets consisted of 267 related genes of 26 ADs (Supplementary Table S1).

### Calculation of Network Similarity Between ADs

We downloaded the information on protein-protein interactions of human genes from the Human Protein Reference Database (HPRD, http://www.hprd.org/) (Peri et al., 2003) and used Cytoscape software (v3.8.2) (Shannon et al., 2003) to construct a human protein-protein interaction network. The topological properties of AD-related genes in this network were computed (Hidalgo et al., 2009; Chavali et al., 2010). The gene set of disease *d* was defined as *G* = {*g*_1_, *g*_2_, *g*_3_, … , *g*_*i*_, … , *g*_*k*_}. In order to assessed the network similarity between ADs, we first calculated the average topological properties of each AD in the network as follows:

$$R=\frac{\sum_{1\leq i\leq k}r_{i}}{k},C=\frac{\sum_{1\leq i\leq k}c_{i}}{k},B=\frac{\sum_{1\leq i\leq k}b_{i}}{k},S=\frac{\sum_{1\leq i\leq k}s_{i}}{k},andH=\frac{\sum_{1\leq i\leq k}h_{i}}{k}$$ (1-5)

where *k* represents the number of genes in *G*, *g*_*i*_ is the *i*th gene of *G*, *r*_*i*_ is the degree of *g*_*i*_, *c*_*i*_ is the clustering coefficient of *g*_*i*_, *b*_*i*_ is the betweenness centrality of *g*_*i*_, *s*_*i*_ is the average shortest path length of *g*_*i*_, *h*_*i*_ is the neighborhood connectivity of *g*_*i*_, *R* is the degree of *d*, *C* is the clustering coefficient of *d*, *B* is the betweenness centrality of *d*, *S* is the average shortest path length of *d*, and *H* is the neighborhood connectivity of *d*. As shown in Figure 1, *d*_1_ and *d*_2_ are two ADs from MeSH, and *G*_1_ and *G*_2_ are gene sets related to *d*_1_ and *d*_2_. The average topological properties of *d*_1_ and *d*_2_ were defined as vector *T*_1_ = {*R*_1_, *C*_1_, *B*_1_, *S*_1_, *H*_1_} and vector *T*_2_ = {*R*_2_, *C*_2_, *B*_2_, *S*_2_, *H*_2_}, respectively. We defined the network similarity (NetSim) score between *d*_1_ and *d*_2_ as the Pearson correlation coefficient (PCC) calculated with *T*_1_ and *T*_2_. The formula that was used as follows:

$$\rho_{T_{1},T_{2}}=\frac{\mathrm{cov}\left(T_{1},T_{2}\right)}{\sigma_{T_{1}},\sigma_{T_{2}}}$$ (6)

where cov(*T*_1_,*T*_2_) is the covariance of variables *T*_1_ and *T*_2_, *σT*_1_ and *σT*_2_ are the standard deviations for *T*_1_ and *T*_2_.

### Calculation of Functional Similarity Between ADs

The data on the functional interactions of genes was downloaded from HumanNet, which is a human gene functional interaction network based on Gene Ontology annotation (Lee et al., 2011). Each interaction in HumanNet has a log likelihood score (LLS) that measures the probability of a functional association between genes (Cheng et al., 2014).

We downloaded LLSs between human genes from HumanNet and normalized the LLSs as follows:

$$LLS_{N}\left(g_{i},g_{j}\right)=\frac{LLS\left(g_{i},g_{j}\right)-LLS_{\min}}{LLS_{\max}-LLS_{\min}}$$ (7)

where *g*_*i*_ and *g*_*j*_ are the *i*th and *j*th gene respectively. *LLS*_*N*_(*g*_*i*_, *g*_*j*_) indicates LLS between *g*_*i*_ and *g*_*j*_ after normalization. *LLS*(*g*_*i*_, *g*_*j*_) indicates LLS between *g*_*i*_ and *g*_*j*_. *LLS*_min_ and *LLS*_max_ are the minimum LLS and the maximum LLS of HumanNet respectively.

The functional similarity (FunSim) score between a pair of genes was defined as follows:

$$FunSim\left(g_{i},g_{j}\right)=\left\{ \begin{array}{l} 1\\ LLS_{N}\\ 0 \end{array}\right.\begin{array}{cc} & \begin{array}{c} i=j\\ i\neq j\\ i\neq j \end{array}\begin{array}{c} and\\ and \end{array} \end{array}\begin{array}{c} e\left(i,j\right)\in \text{E}\left(HumanNet\right)\\ e\left(i,j\right)\notin \text{E}\left(HumanNet\right) \end{array}$$ (8)

where *e*(*i*, *j*) represents the interaction edge between *g*_*i*_ and *g*_*j*_. E(*HumanNet*) is a set including all the edges of HumanNet.

Next, we defined the functional association between a gene *g* and a gene set *G* = {*g*_1_, *g*_2_, *g*_3_, … , *g*_*i*_, … , *g*_*k*_} as follows:

$$F_{G}\left(g\right)=\underset{1\leq i\leq k}{\max}\left(FunSim\left(g,g_{i}\right)\right),g_{i}\in G$$ (9)

where *k* represents the number of genes in *G*, *g*_*i*_ is the *i*th gene of *G*.

*G*_1_ = {*g*_11_, *g*_12_, … , *g*_1*i*_, … , *g*_1*m*_} and *G*_2_ = {*g*_21_, *g*_22_, … , *g*_2*j*_, … , *g*_2*n*_} are gene sets related to *d*_*1*_ and *d*_*2*_ respectively. *m* is the number of genes in *G*_1_, and *n* is the number of genes in *G*_2_. We defined FunSim score of *d*_1_ and *d*_2_ as follows:

$$FunSim\left(d_{1},d_{2}\right)=\frac{\sum_{1\leq i\leq m}F_{G_{2}}\left(g_{1i}\right)+\sum_{1\leq j\leq n}F_{G_{1}}\left(g_{2j}\right)}{m+n},g_{1i}\in G_{1},g_{2j}\in G_{2}$$ (10)

### Calculation of Semantic Similarity Between ADs

Semantic similarity is a method to measure the closeness between two terms, according to a given ontology (Zhang and Lai, 2015; Zhang and Lai, 2016; Del Prete et al., 2018). The Resnik method was applied to our study. The human disease terms were obtained from the Human Disease Ontology (DO, http://www.disease-ontology.org) (Schriml et al., 2019). The DO includes the breadth of common and rare diseases, organized as a directed acyclic graph in which a term represents a DO term and an edge represents an “IS_A” relationship between diseases. The information content (IC) of each DO term could be calculated as follows:

$$IC\left(d\right)=-\log\left(\frac{n}{N}\right)$$ (11)

where *d* is a disease term of DO, *n* is the number of genes related to *d*, and *N* is the total number of genes related to DO. As shown in Figure 1, *d*_1_ and *d*_2_ are two AD terms of DO, and *d*_*MICA*_ is the most informative common ancestor (MICA) of *d*_1_ and *d*_2_. The MICA means the ancestor that has the maximum IC among all the common ancestors between terms of ontology. And we defined the semantic similarity (SemSim) score of *d*_1_ and *d*_2_ as follows:

$$SemSim\left(d_{1},d_{2}\right)=IC\left(d_{MICA}\right)$$ (12)

### Calculation of Integrated Similarity Between ADs

To identify more reliable pairs of similar ADs, we integrated above three kinds of similarity (NetSim, FunSim, and SemSim) scores to comprehensively determine the levels of similarity between ADs as follows:

$$IntegratedSim\left(d_{1},d_{2}\right)=\sqrt[{3}]{NetSim\left(d_{1},d_{2}\right)\cdot FunSim\left(d_{1},d_{2}\right)\cdot SemSim\left(d_{1},d_{2}\right)}$$ (13)

where *d*_1_ and *d*_2_ are two AD terms of MeSH.

### Functional Analysis of Related Genes of Similar ADs

Functional enrichment analysis of Gene Ontology (GO) and Kyoto encyclopedia of genes and genomes (KEGG) for related genes of similar ADs was performed to infer potential biological processes and pathways using the DAVID Bioinformatics Tool (http://david.abcc.ncifcrf.gov/, version 6.7) (Huang Da et al., 2009). The *p*-values for the biological processes and pathways were adjusted for false discovery rate (FDR) by the Benjamini-Hochberg method. The biological processes and pathways with FDR less than 0.05 were considered statistically significant functional categories.

### Cluster Analysis for ADs Based on Three Kinds of Similarity

To determine whether multiple ADs had high genetic similarity, hierarchical clustering was performed on the integrated similarity scores between 26 ADs based on the Euclidean distance. The *d*_1_ and *d*_2_ are two diseases of 26 ADs. We defined the Euclidean distance between *d*_1_ and *d*_2_ as follows:

$$E\left(d_{1},d_{2}\right)=\sqrt{\sum_{1\leq i\leq 26}{\left(x_{1i}-x_{2i}\right)}^{2}}$$ (14)

where *x*_1*i*_ is the integrated similarity score between *d*_1_ and *i*th disease of 26 ADs, *x*_2*i*_ is the integrated similarity score between *d*_2_ and *i*th disease of 26 ADs. The 26 ADs were clustered based on the Euclidean distances between diseases and there were shorter Euclidean distances between ADs belonging to the same disease cluster.

## Results

### Identification of Potential Pairs of Similar ADs

**FIGURE 2:**
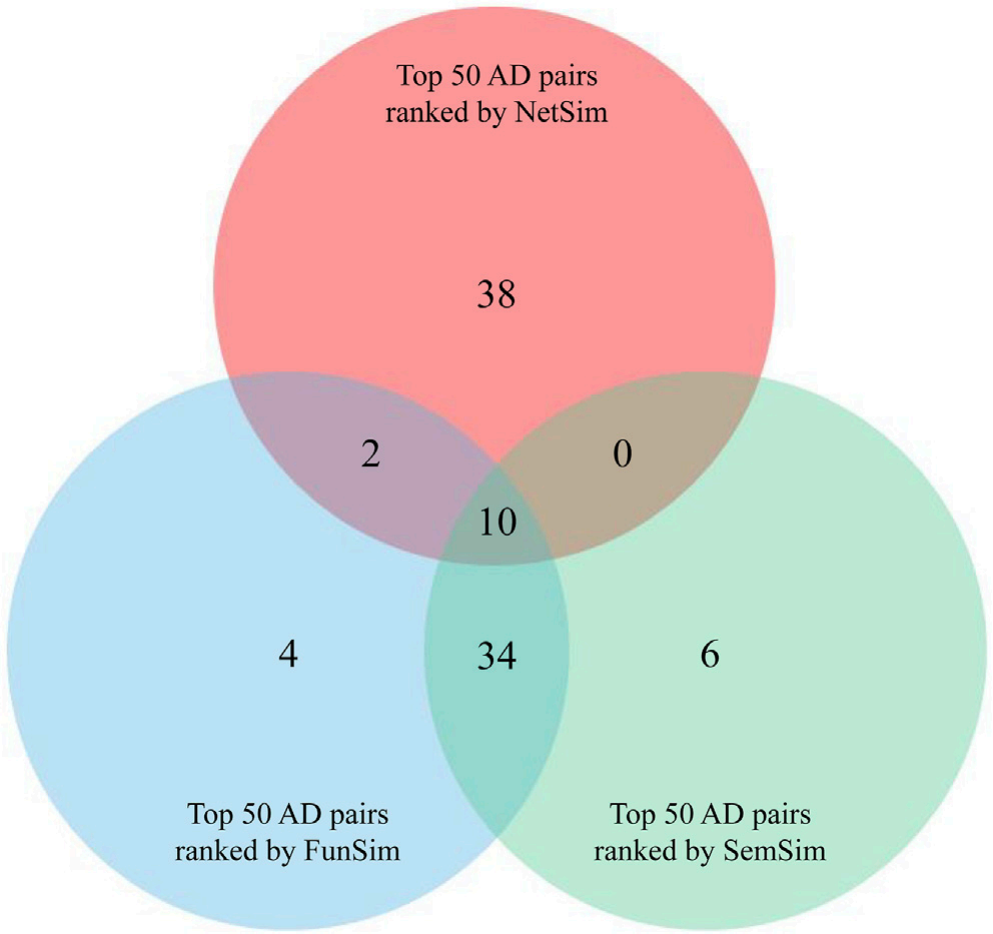
Venn diagram analysis of three groups of AD pairs ranked by NetSim, FunSim, and SemSim respectively.

To identify pairs of similar diseases in genetics, we calculated similarity between 26 ADs based on three measurements. All of AD pairs were sorted in descending according to their scores of NetSim, FunSim, and SemSim. To enhance the reliability of the study, we selected ten AD pairs from intersection of top 50 AD pairs with the highest NetSim score, top 50 AD pairs with the highest FunSim score, and top 50 AD pairs with the highest SemSim score for further analysis (Figure 2 and Supplementary Tables S2–4), which were considered as potential pairs of similar ADs.

**FIGURE 3:**
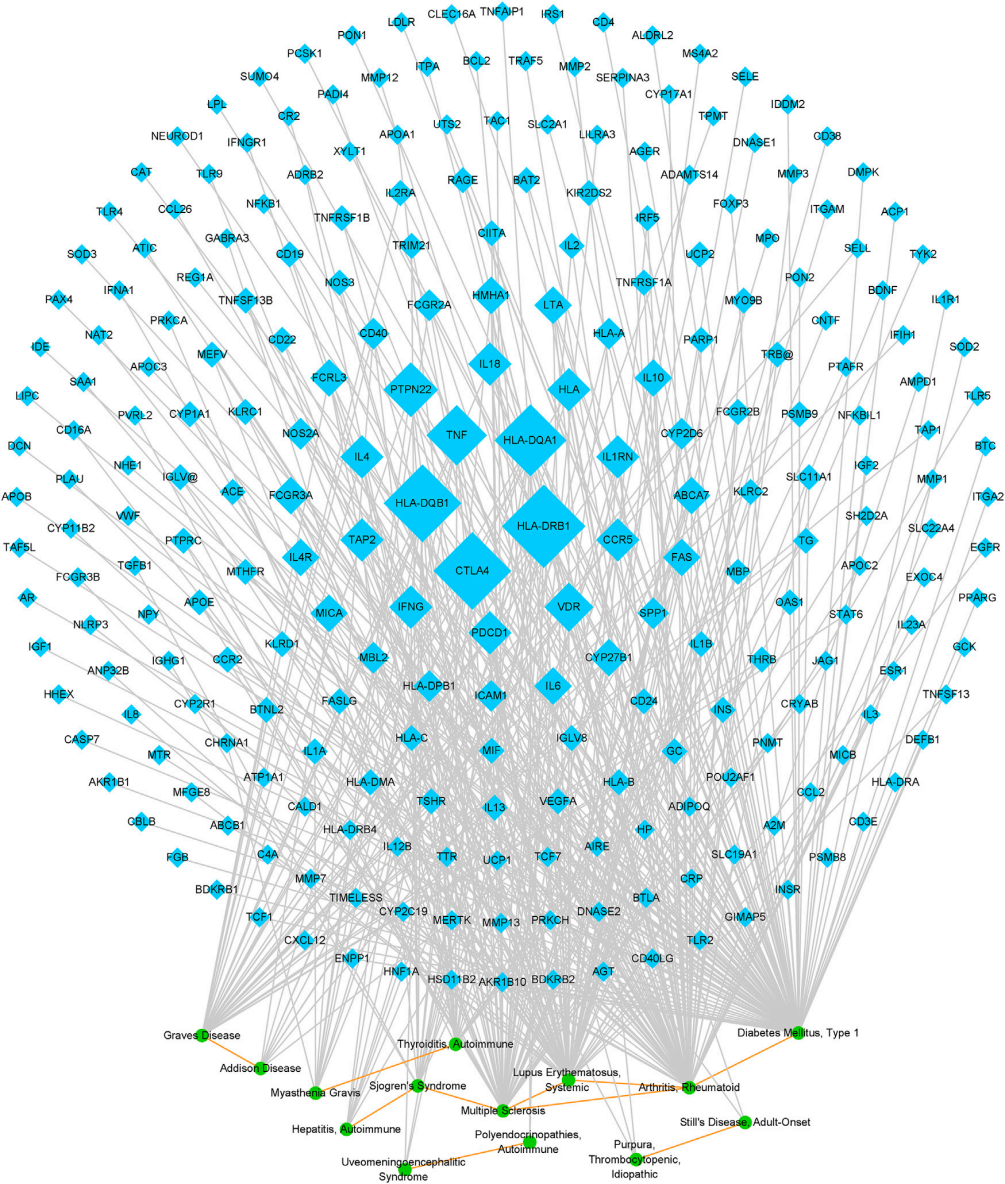
Network on potential pairs of similar ADs and AD-gene relationships. The blue nodes represent genes, and the size of these nodes corresponds to the node degree. The green nodes represent ADs. The gray edges represent disease-gene relationships, and the orange edges represent potential AD similarity relationships.

Further, a network was generated on the ten AD pairs and their related genes, including 14 ADs and 247 genes (Figure 3). The topological properties of the network were investigated. We extracted 35 genes each of whose degree was greater than or equal to three from the network as the possible AD relevant genes. The top five genes regarding degree were *HLA-DRB1*, *HLA-DQB1*, *CTLA4*, *HLA-DQA1*, and *TNF*, indicating that these genes were critical in multiple ADs. Previous researches have demonstrated that these genes are associated with many ADs and exert various functions in human autoimmune disorders. Besides, we ascertained that RA and multiple sclerosis (MS) involved more similarity relationships than other AD in this network, implying that the pathogenesis of RA and MS might exist in most of ADs from the network. What’s more, a pair of diseases with higher number of shared genes, suggesting they likely have higher genetic similarity. Thus, the ten potential pairs of similar ADs were ranked by their number of shared genes and shown in Table 1. And the top-rank disease pair is RA and SLE, followed by T1D and RA, and RA and MS.

**TABLE 1:** The ten potential pairs of similar ADs ranked by number of shared genes.

Rank	Autoimmune disease	Autoimmune disease	Number of shared gene
1	Arthritis, Rheumatoid	Lupus Erythematosus, Systemic	40
2	Diabetes Mellitus, Type 1	Arthritis, Rheumatoid	28
3	Arthritis, Rheumatoid	Multiple Sclerosis	23
4	Lupus Erythematosus, Systemic	Multiple Sclerosis	20
5	Multiple Sclerosis	Sjogren’s Syndrome	9
6	Myasthenia Gravis	Thyroiditis, Autoimmune	7
7	Addison Disease	Graves Disease	6
8	Hepatitis, Autoimmune	Sjogren’s Syndrome	3
9	Polyendocrinopathies, Autoimmune	Uveomeningoencephalitic Syndrome	2
10	Purpura, Thrombocytopenic, Idiopathic	Still’s Disease, Adult-Onset	1

### Functional Implication of the Genes Related to Multiple ADs

**FIGURE 4:**
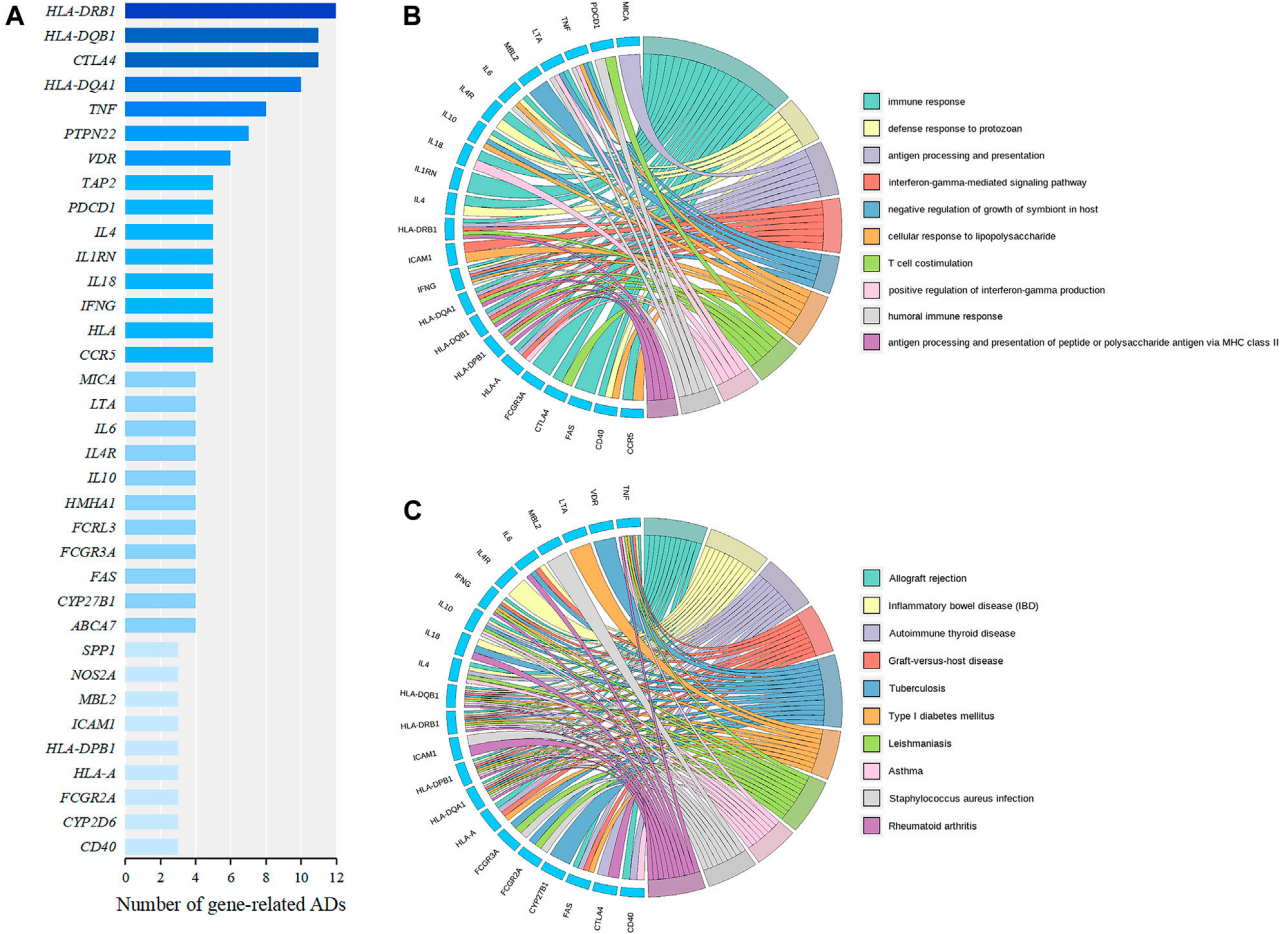
Identification and functional enrichment analysis of AD relevant genes. **(A)** The genes correlated with at least three ADs derived from the AD similarity network, which are ranked by the number of related diseases. **(B)** Circos plot of top ten significant GO terms in the BP. **(C)** Circos plot of top ten significant KEGG pathways. The genes are displayed on the left half of the circos plots. The right half represents different GO terms or KEGG pathways with different colors. A gene is linked to a certain GO term or KEGG pathway by the colored bands.

To explore common genetic mechanisms of a variety of ADs, we performed functional enrichment analysis of GO and KEGG for the 35 AD relevant genes (Figure 4A). The cutoff criterion was a FDR less than 0.05. The top ten significant GO terms in the BP were mainly associated with immune response, antigen processing and presentation, and interferon gamma (IFNγ)-related functions (Figure 4B). Notably, *IFNG* was involved in the top three GO terms and was defined as a hub gene. *IFNG* can encode IFNγ that is a cytokine that is critical for innate and adaptive immunity against viral, bacterial, and protozoan infections. And aberrant IFNγ expression is associated with a number of ADs, such as RA and SLE (Hu and Ivashkiv, 2009; Barrat et al., 2019). The top ten significant KEGG pathways contained four AD-correlated pathways, such as “inflammatory bowel disease (IBD),” “autoimmune thyroid disease,” “type 1 diabetes mellitus,” and “rheumatoid arthritis,” which illustrated that the 35 genes might induce the initiation and development of multiple ADs (Figure 4C). *HLA-DQB1*, *HLA-DRB1*, *HLA-DPB1*, *HLA-DQA1* were involved in all of the top ten KEGG pathways and were defined as hub genes. The four genes belonged to HLA class II alleles which were suggested to contribute to the susceptibility and resistance to ADs (Wieber et al., 2021).

### Identification of Significant Pairs of Similar ADs

To identify more reliable pairs of similar ADs, we integrated the three measurements to compute integrated similarity scores between 26 ADs (see Materials and Methods). All of AD pairs were sorted in descending according to their integrated similarity scores. And the top ten AD pairs were extracted for further analysis (Table 2). We found that the ten AD pairs contained three potential pairs of similar ADs consisting of RA and SLE, myasthenia gravis (MG) and autoimmune thyroiditis (AIT), and autoimmune polyendocrinopathies (AP) and uveomeningoencephalitic syndrome (Vogt-Koyanagi-Harada syndrome, VKH) which were defined as the significant pairs of similar ADs.

**TABLE 2:** Top ten pairs of ADs ranked by integrated similarity scores.

Rank	Autoimmune disease	Autoimmune disease	Integrated similarity score
1	Polyendocrinopathies, Autoimmune	Uveomeningoencephalitic Syndrome	0.704362256
2	Thyroiditis, Autoimmune	Graves Disease	0.605323256
3	Myasthenia Gravis	Thyroiditis, Autoimmune	0.540495323
4	Addison Disease	Hepatitis, Autoimmune	0.53677382
5	Uveomeningoencephalitic Syndrome	Addison Disease	0.534304723
6	Arthritis, Rheumatoid	Lupus Erythematosus, Systemic	0.527496589
7	Pemphigoid, Bullous	Polyendocrinopathies, Autoimmune	0.52566119
8	Hepatitis, Autoimmune	Myasthenia Gravis	0.507462033
9	Anemia, Hemolytic, Autoimmune	Purpura, Thrombocytopenic, Idiopathic	0.499501
10	Guillain-Barre Syndrome	Still’s Disease, Adult-Onset	0.499448795

### Functional Analysis of Related Genes of Significant Pairs of Similar ADs

**FIGURE 5:**
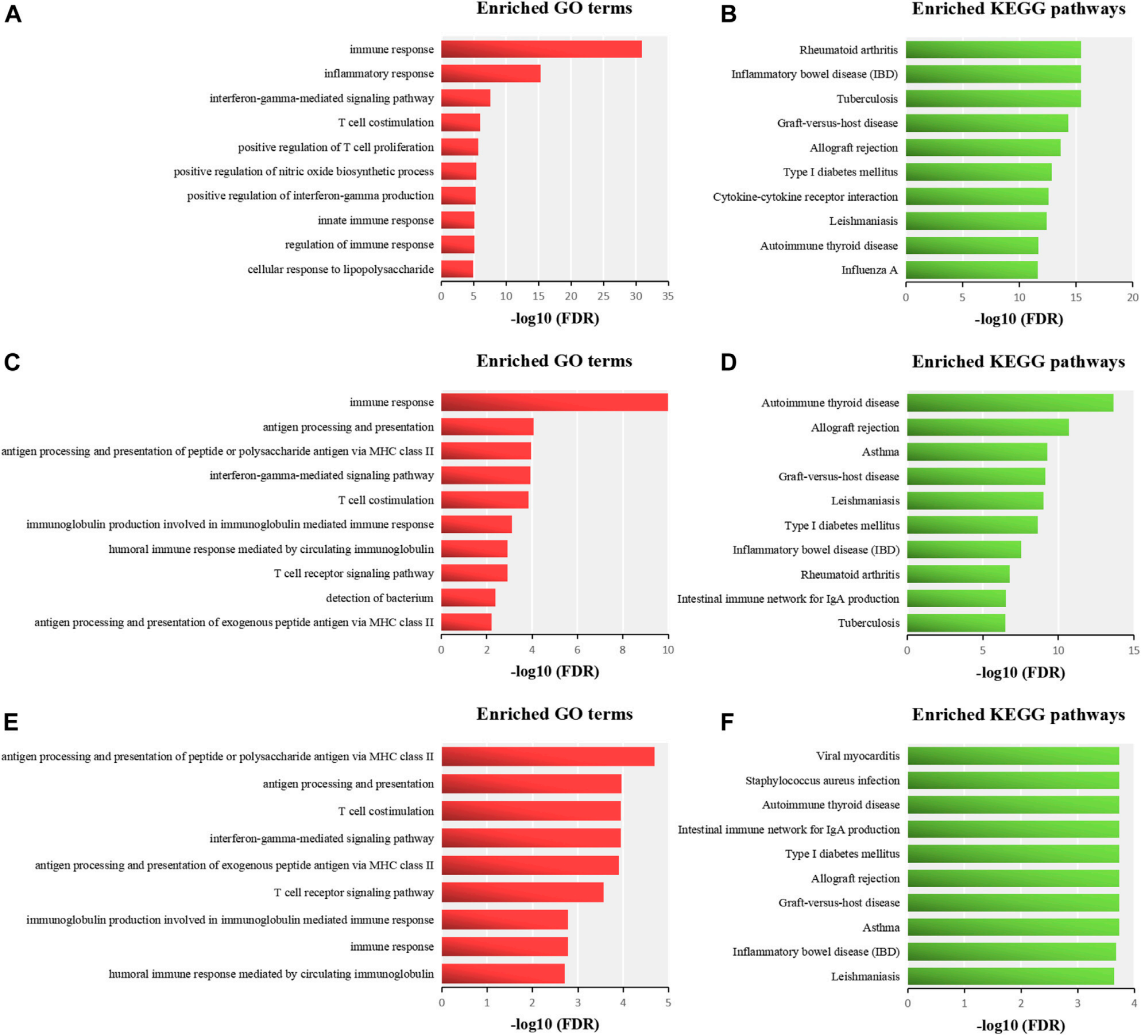
Functional enrichment analysis of related genes of RA and SLE **(A-B)**, MG and AIT **(C-D)**, and AP and VKH **(E-F)**. Enriched functional terms are sorted in descending order according to their–log10 (FDR), and the top ten significant GO terms in the BP and KEGG pathways of each significant pair of similar ADs are used for further analysis.

To reveal the underlying mechanisms shared by two similar ADs, we performed functional enrichment analysis of related genes of RA and SLE, MG and AIT, and AP and VKH. The cutoff criterion was a FDR less than 0.05. The related genes of RA and SLE were significantly enriched in GO terms mainly involved in immune response, inflammatory response, and IFNγ. The significant enriched pathways including RA, inflammatory bowel disease (IBD), tuberculosis, etc (Figures 5A,B). The related genes of MG and AIT were mainly related to immune response (GO), antigen processing and presentation (GO), autoimmune thyroid disease (AITD) (KEGG), and allograft rejection (KEGG) (Figures 5C,D). Moreover, the related genes of AP and VKH were mainly associated with the antigen processing and presentation in GO and some pathways such as viral myocarditis, *Staphylococcus aureus* infection, AITD, intestinal immune network for IgA production, etc (Figures 5E,F).

### Hierarchical Clustering Result of 26 ADs Based on Three Similarity Measurements

**FIGURE 6:**
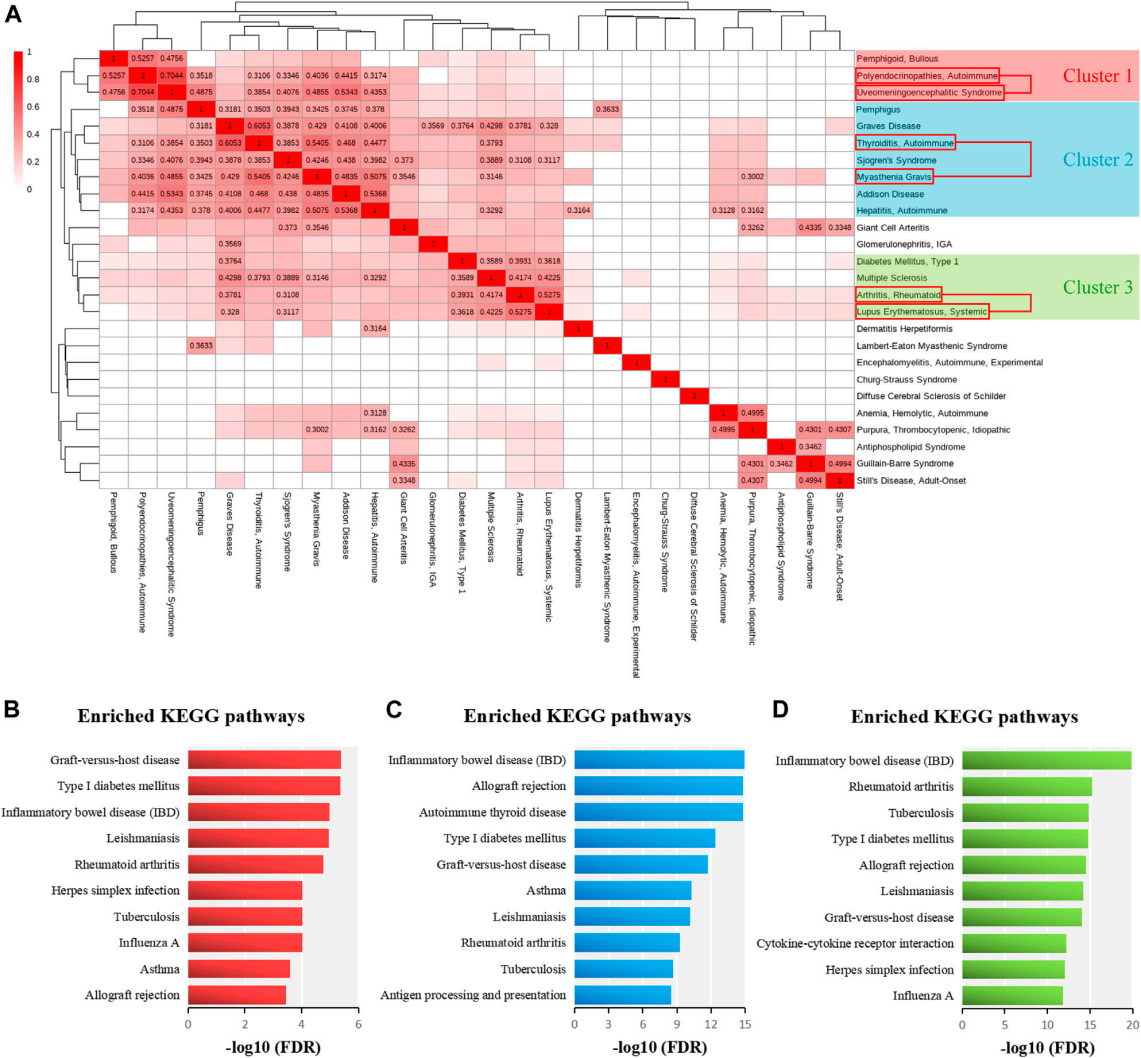
Cluster analysis for 26 ADs and pathway enrichment analysis for ADs of each significant cluster. **(A)** The clustering heatmap illustrating the classification of 26 ADs based on Euclidean distances and integrated similarity scores between 26 ADs. The similarity values, which are greater than 0.3, are marked in the matrix. **(B)** The top ten significant KEGG pathways of related genes of ADs of cluster one ranked by–log10 (FDR). **(C)** The top ten significant KEGG pathways of related genes of ADs of cluster two ranked by–log10 (FDR). **(D)** The top ten significant KEGG pathways of related genes of ADs of cluster three ranked by–log10 (FDR).

To determine whether there was high genetic similarity among multiple ADs, we applied hierarchical clustering to the integrated similarity matrix of 26 ADs. The disease clusters consisting of AD pairs with integrated similarity scores greater than 0.3 were considered to be significant. As shown in Figure 6A, three significant disease groups were identified from the 26 ADs, and the ADs of each disease group might have high genetic similarity. We found that the three significant pairs of similar ADs were located in three different clusters, respectively. In the cluster one, bullous pemphigoid might be similar to AP and VKH. The ADs of cluster one are involved in endocrine autoimmunity. For instance, bullous pemphigoid has been proved to be related to immunodysregulation polyendocrinopathy enteropathy X-linked syndrome (Mcginness et al., 2006). In the cluster two, pemphigus, Graves disease, Sjogren’s syndrome, Addison Disease, and autoimmune hepatitis might be similar to MG and AIT. The ADs of cluster two contain the main AITDs (AIT and Graves disease) and frequent ADs involved in PolyA (AIT, Graves disease, and Sjogren’s syndrome) (Amador-Patarroyo et al., 2012; Botello et al., 2020). In the cluster three, T1D and MS might be similar to RA and SLE. The ADs of cluster three are all chronic inflammatory ADs and share multiple genetic susceptibility loci (Richard-Miceli and Criswell, 2012). Then, we performed pathway enrichment analysis of the related genes of ADs of three significant clusters. The related genes of ADs of three disease clusters were significantly enriched in pathways of T1D and IBD (Figures 6B–D). Therefore, we infer that T1D and IBD can participate in PolyA in various AD patients.

Next, we detected the causal genes in common among the ADs which belonged to the same significant disease cluster, and these genes could be used for PolyA research. As shown in Table 3, the ADs of cluster one shared one gene (*HLA-DQA1*); the ADs of cluster two shared two genes (*HLA-DQB1* and *HLA-DRB1*); the ADs of cluster three shared eight genes (*TNF*, *HLA-DRB1*, *PDCD1*, *PTPN22*, *CCR5*, *IL6*, *HLA-DQB1*, and *CTLA4*). Identification of these genes will contribute to the discovery of novel prognostic, diagnostic, and therapeutic markers and justification of drug repurposing for ADs.

**TABLE 3:** The shared causal genes among the ADs which belong to the same significant disease cluster.

Disease cluster	Shared gene
Cluster one	*HLA-DQA1*
Cluster two	*HLA-DQB1*, *HLA-DRB1*
Cluster three	*TNF*, *HLA-DRB1*, *PDCD1*, *PTPN22*, *CCR5*, *IL6*, *HLA-DQB1*, *CTLA4*

Italics refers to gene symbols (gene names).

## Discussion

During the past years, numerous studies have confirmed that different ADs are similar in various aspects. Nevertheless, these studies just focused on several ADs, and lacking a comprehensive analysis on similarity between ADs from the perspective of genetics. To date, various disease similarity methods have been developed (Dozmorov, 2019). In this study, we calculated the similarity scores between 26 ADs by means of three similarity measurements. To ensure the accuracy of the subsequent analysis, we combined the results of NetSim, FunSim, and SemSim to evaluate all the AD pairs. We found ten potential pairs of similar ADs that were utilized to form a network containing an overall insight of the information about AD-AD relationships and AD-gene relationships, which provided essential clues to understand the mechanisms shared by multiple ADs. Based on the AD pairs in this network, we detected three significant pairs of similar ADs (RA and SLE, MG and AIT, and AP and VKH), and then investigated the shared functional terms for each significant AD pair. We also employed cluster analysis on the integrated similarity matrix of 26 ADs to acquire some other ADs which were similar to significant AD pairs in genetics, and identified the risk genes which belonged to the same disease cluster. These results still need to be verified by more studies, but we hope that our observations can help researchers to dissect the complex pathogenesis of ADs.

By the functional enrichment analysis of 35 AD relevant genes, we mainly focused on GO terms involved in immune response, antigen processing and presentation, and IFNγ. The immune response is how the immune system defends against foreign invaders, such as bacteria or viruses (Chaplin, 2010). ADs are triggered by aberrant immune response which damages healthy body part and is influenced by a large number of genes (Hill et al., 2008; Gregersen and Olsson, 2009). We concluded that the 35 genes might trigger a variety of ADs. On the other hand, the dysfunction of antigen processing and presentation might influence the emergence of ADs (Ritz and Seliger, 2001). In human bodies, antigens are processed into peptides of a certain length in association with major histocompatibility complex (MHC) molecules. T cells are capable of recognizing these fragmented peptides bound to the MHC to initiate immune responses (Purcell et al., 2016; Kelly and Trowsdale, 2019; Kotsias et al., 2019). As different ADs share the characteristic that risk is conferred by genes encoded within the MHC locus, antigen presentation generally seems to be crucial in ADs (Riedhammer and Weissert, 2015). For example, processing and presentation of self-antigens by different antigen presenting cells may result in MS (Stoeckle and Tolosa, 2010). Ultimately, IFNγ is a pleiotropic cytokine secreted by immune cells and plays a critical role in innate and adaptive immunity (Tau and Rothman, 1999; Schoenborn and Wilson, 2007). Abnormal IFNγ expression is correlated with considerable number of ADs. Although IFNγ can mediate clearance of pathogenic insults, chronic exposure to IFNγ is thought to cause many ADs, such as RA and SLE (Nielen et al., 2004; Lu et al., 2016). And the complex role of IFNγ in ADs also has important therapeutic implications. Above evidences demonstrate that the three function aspects play important roles in AD-related mechanisms.

With regard to the three significant pairs of similar ADs, several studies have confirmed these similarity relationships. For example, (Wang et al., 2020) found that familial RA, SLE, and primary Sjögren’s syndrome shared common genetic characteristics, and the genetic variations in T cell receptor signaling pathway genes which might become novel molecular targets for therapeutic interventions for the three ADs. (Liu et al., 2019) found that T cell receptor could become a promising diagnostic marker for RA and SLE. In addition, previous studies have confirmed that MG and AIT are similar in many aspects (Marino et al., 1997; Lopomo and Berrih-Aknin, 2017), and AIT frequently accompanies MG (Mao et al., 2011; Kubiszewska et al., 2016). The two diseases are both organ-specific ADs with a clear pathogenic effect of antibodies. Meanwhile, MG and AIT share the same predisposing genes (such as *PTPN22*, *CTLA4*, and *HLA*) and pathological mechanisms (such as T-cell immune-mediated mechanisms). Thus, we infer that AD genetic similarity research can help to explain the similar phenotypic and clinical features between ADs. These reports are consistent with our current results. Experimental studies on these AD pairs are desperately needed to provide important information to understand their intrinsic mechanisms. And further validation of these disease relationships in clinical trials will be a better option to turn them into clinical practice. Besides, the results of pathway enrichment analysis of related genes of significant pairs of similar ADs exposed possible PolyA. For example, the related genes of RA and SLE were enriched in pathways of IBD, T1D, and AITD. It was reported that AIT frequently coexisted with RA and SLE (Ordonez-Canizares et al., 2020). Another study showed that AITD, RA, SLE, and IBD were observed in Sjögren’s syndrome patients with PolyA (Amador-Patarroyo et al., 2012). And the related genes of MG and AIT were enriched in pathways of T1D, IBD, and RA. Previous study found that the latent and overt PolyA in patients with AITD were associated with gastrointestinal, endocrinological, rheumatological, dermatological, and neurological ADs (Botello et al., 2020). The PolyA is not uncommon and multiple ADs that coexist in a single patient may share the same etiopathogenesis. Some genetic studies on ADs ignored the coexistence of other autoimmune conditions by implementing anachronistic nomenclature (i.e., primary or secondary ADs) (Rojas-Villarraga et al., 2012). We hope that researchers can take in account PolyA and concern whether or not patients have latent or overt PolyA in AD study.

With regard to the result of cluster analysis on 26 ADs, hitherto, a lot of reports have confirmed our viewpoint. For example, for the disease cluster two, a recent study found that chemokines were associated with the early phases of the autoimmune response in AIT, Graves disease, and Addison disease (Fallahi et al., 2020). For the disease cluster three, another study found major common gene expression changes at the target tissues of T1D, MS, RA, and SLE (Szymczak et al., 2021).

This study predicted AD pairs and clusters with high genetic similarity, as well as potential risk genes, biological processes, and pathways involved in multiple types of ADs. Despite the two diseases of a certain AD pair with high similarity score have different phenotypic or clinical features, they are likely to have similar or the same ways to elicit autoimmune responses in the human body. Consequently, we reason that similar ADs in genetics can be treated with similar therapeutics and drugs. We hope that these findings can aid in elucidating AD mechanisms, and provide more references for researchers.

## Data Availability

The original contributions presented in the study are included in the article/Supplementary Material, further inquiries can be directed to the corresponding authors.
