# Supplementary material for: Calculation of Similarity Between 26 Autoimmune Diseases Based on Three Measurements Including Network, Function, and Semantics
Source: Front Genet. 2021 Nov 11;12:758041. doi: 10.3389/fgene.2021.758041 (PMC8632457; doi:10.3389/fgene.2021.758041)
Supplement: Supplementary file 1 [file Table1.DOCX]

**Supplementary Table 1** Related genes of 26 autoimmune diseases from Genetic Association Database.

| **Autoimmune disease** | **Related gene** |
| --- | --- |
| Addison Disease | *VDR, INS, CYP2D6, CYP27B1, CTLA4, HLA-DQB1, HLA-DRB1, MICA, HLA-DQA1* |
| Anemia, Hemolytic, Autoimmune | *CTLA4* |
| Churg-Strauss Syndrome | *MPO* |
| Antiphospholipid Syndrome | *FCGR2A, SELPLG, SERPINE1, HLA-DMA, F2* |
| Arthritis, Rheumatoid | *CYP17A1, MMP2, ITPA, IFNG, MEFV, TNFSF13B, IL4, CCL26, NAT2, IL4R, PLAU, KIR2DS2, IRF5, MYO9B, FCGR2A, LTA, MMP7, TNF, MMP13, BTLA, SLC19A1, MICB, VEGFA, HLA-DMA, IL3, IL23A, EXOC4, SLC22A4, IL1A, HLA-DRB1, PDCD1, MMP3, SERPINA3, ICAM1, IGLV@, CYP1A1, MBL2, CD19, TNFRSF1B, ATIC, RAGE, BAT2, CYP11B2, NLRP3, FCRL3, TRB@, HMHA1, PTPN22, SLC11A1, FCGR2B, PRKCH, IL10, A2M, ABCA7, TNFRSF1A, CIITA, CCR5, CD22, SPP1, MMP1, AMPD1, MTHFR, HLA-DQA1, KLRD1, IL6, TRAF5, MMP12, HLA-DQB1, PADI4, TAP2, MIF, MICA, SAA1, IGLV8, FCGR3A, NOS2A, IL1B, MTR, KLRC2, ABCB1, TIMELESS, IL18, HLA, IL1RN, CTLA4, PARP1, IL2, FAS, ESR1, KLRC1* |
| Sjogren's Syndrome | *IFNG, TGFB1, HLA-DRB1, HMHA1, HLA-A, IGHG1, IL10, ABCA7, CCR5, HLA-DQA1, TRIM21, HLA-DQB1, TAP2, CTLA4* |
| Still's Disease, Adult-Onset | *FCGR3A, IL18* |
| Diffuse Cerebral Sclerosis of Schilder | *PLP1* |
| Multiple Sclerosis | *VDR, MEFV, FASLG, CD24, HMHA1, PTPN22, APOE, CTLA4, IL2, FAS, POU2AF1, PNMT, IFNG, IL4, CRYAB, HLA-DPB1, FCRL3, JAG1, CIITA, HLA-DQB1, APOC2, IL1RN, SH2D2A, IL4R, BTNL2, PTAFR, OAS1, TNF, CNTF, HLA-DRB1, PDCD1, CCR2, MPO, MBP, HLA-A, ADAMTS14, ABCA7, CCR5, SPP1, LILRA3, IL1B, TAC1, APOA1, ADRB2, GABRA3, PRKCA, HLA-DQA1, IL6, PVRL2, UCP2, PTPRC* |
| Myasthenia Gravis | *FCGR2A, TNF, IL1A, HLA-DRB1, CD40, PTPN22, CHRNA1, HLA-DQA1, HLA-DQB1, CTLA4* |
| Lambert-Eaton Myasthenic Syndrome | *HLA-B* |
| Encephalomyelitis, Autoimmune, Experimental | *CD24* |
| Guillain-Barre Syndrome | *FCGR2A, FCGR3A* |
| Uveomeningoencephalitic Syndrome | *HLA-DRB1, HLA-DQA1, HLA-DQB1* |
| Giant Cell Arteritis | *IFNG, IL4, FCGR2A, VEGFA, HLA-DRB1, IL10, NOS3, FCGR3A* |
| Dermatitis Herpetiformis | *TNF* |
| Diabetes Mellitus, Type 1 | *VDR, ACE, PON1, SUMO4, NEUROD1, TLR4, SOD3, IFNG, IL4, IDE, DCN, TAF5L, IGF1, CASP7, IL4R, CBLB, HLA-C, HLA-DPB1, BDKRB1, KIR2DS2, CXCL12, LTA, HNF1A, OAS1, BDKRB2, INS, TLR2, TNF, INSR, PSMB8, VEGFA, HLA-DRA, GCK, ITGA2, SOD2, ACP1, IDDM2, HLA-DRB1, PDCD1, CCR2, ALDRL2, CD4, IRS1, CLEC16A, ICAM1, LDLR, PCSK1, MBL2, LPL, CAT, IL2RA, PAX4, RAGE, LIPC, APOB, BAT2, AR, HHEX, AKR1B1, FGB, TCF1, ENPP1, PTPN22, HSD11B2, AKR1B10, HLA-A, AGT, CYP2D6, IL13, SLC11A1, CD3E, DEFB1, PPARG, IL10, BTC, TNFRSF1A, IL1R1, DMPK, IGF2, CCR5, NFKBIL1, MTHFR, HLA-DQA1, CYP27B1, NOS3, IL6, PON2, FOXP3, HLA-DQB1, TAP2, AGER, SLC2A1, UTS2, XYLT1, NFKB1, MICA, REG1A, APOC3, NOS2A, NHE1, VWF, IL18, NPY, UCP2, CYP2R1, PSMB9, HLA, ATP1A1, CALD1, CTLA4, IL12B, UCP1, TCF7, HP, ADIPOQ* |
| Glomerulonephritis, IGA | *PON1, ACE, IFNG, IL4, selectin, IGHMBP2, MUC20, VEGFA, TGFB1, ITGA2, ICAM1, SELP, PIGR, CYP11B2, MBP, AGT, SCGB1A1, SERPINB7, HLA-DQB, TRA@, NPHS1, HLA-DQB1, SELL, IL1B, SELE, FCAR, C1GALT1, HLA, IL1RN, ADD1, CD14* |
| Graves Disease | *VDR, IFNG, IL4, IL4R, BTNL2, GC, TNF, STAT6, THRB, HLA-DRB1, PDCD1, ICAM1, TSHR, CD40, HLA-B, IL2RA, TAP1, FCRL3, TG, PTPN22, CYP2D6, IL13, HLA-DQA1, IFIH1, CYP27B1, HLA-DQB1, SELL, TAP2, IL18, PSMB9, HLA, IL1RN, CTLA4* |
| Hepatitis, Autoimmune | *VDR, HLA, CTLA4, FAS, HLA-DQB1, TPMT, TNF, HLA-DRB1* |
| Lupus Erythematosus, Systemic | *VDR, ACE, BDNF, ITGAM, DNASE1, TNFSF13B, FASLG, BCL2, HLA-C, HLA-DPB1, CR2, IFNGR1, IRF5, MYO9B, LTA, FCGR2A, TNF, TLR9, HLA-DMA, IFNA1, CD16A, HLA-DRB1, PDCD1, IGLV@, CYP1A1, MBL2, CD19, TNFRSF1B, CD24, FCGR3B, ANP32B, IL8, TRB@, HMHA1, MBP, PTPN22, FCGR2B, MFGE8, C4A, CYP2C19, IL10, MERTK, DNASE2, ABCA7, CCR5, CRP, CCL2, CD22, SPP1, CD40LG, GIMAP5, TRIM21, NOS3, KLRD1, IL6, HLA-DQB1, TNFSF13, TAP2, MIF, EGFR, MICA, IGLV8, TLR5, TYK2, FCGR3A, NOS2A, CD38, SELE, MS4A2, KLRC2, IL18, HLA, IL1RN, APOE, CTLA4, PARP1, PTPRC, FAS, TNFAIP1, KLRC1* |
| Pemphigoid, Bullous | *IL1B, HLA-DQA1* |
| Pemphigus | *DSG1, HLA-DRB1, HLA-B, HLA-DQB1, TAP2, DSG3* |
| Polyendocrinopathies, Autoimmune | *HLA-DRB1, HLA-DQA1* |
| Purpura, Thrombocytopenic, Idiopathic | *LTA, FCGR3A, CTLA4* |
| Thyroiditis, Autoimmune | *HLA-DRB4, TTR, TG, PTPN22, CYP27B1, CTLA4, AIRE, IL4, THRB, CD40, FCRL3, HLA-DQB1, IL1RN, GC, TNF, HLA-DRB1, HLA-B, TSHR, HLA-DQA1* |
