# Supplementary material for: Calculation of Similarity Between 26 Autoimmune Diseases Based on Three Measurements Including Network, Function, and Semantics
Source: Front Genet. 2021 Nov 11;12:758041. doi: 10.3389/fgene.2021.758041 (PMC8632457; doi:10.3389/fgene.2021.758041)
Supplement: Supplementary file 2 [file Table2.DOCX]

**Supplementary Table 2** Top 50 pairs of autoimmune diseases ranked by network similarity scores.

| **Rank** | **Autoimmune disease** | **Autoimmune disease** | **NetSim score** |
| --- | --- | --- | --- |
| 1 | Multiple Sclerosis | Diabetes Mellitus, Type 1 | 0.999950593 |
| 2 | Lupus Erythematosus, Systemic | Diabetes Mellitus, Type 1 | 0.999916321 |
| 3 | Thyroiditis, Autoimmune | Myasthenia Gravis | 0.99990849 |
| 4 | Multiple Sclerosis | Lupus Erythematosus, Systemic | 0.999892701 |
| 5 | Still's Disease, Adult-Onset | Myasthenia Gravis | 0.999816669 |
| 6 | Lupus Erythematosus, Systemic | Arthritis, Rheumatoid | 0.999689333 |
| 7 | Lambert-Eaton Myasthenic Syndrome | Hepatitis, Autoimmune | 0.999678292 |
| 8 | Purpura, Thrombocytopenic, Idiopathic | Graves Disease | 0.999669003 |
| 9 | Uveomeningoencephalitic Syndrome | Polyendocrinopathies, Autoimmune | 0.99965461 |
| 10 | Thyroiditis, Autoimmune | Still's Disease, Adult-Onset | 0.99954767 |
| 11 | Diabetes Mellitus, Type 1 | Arthritis, Rheumatoid | 0.999523147 |
| 12 | Sjogren's Syndrome | Lambert-Eaton Myasthenic Syndrome | 0.999509268 |
| 13 | Giant Cell Arteritis | Addison Disease | 0.999463785 |
| 14 | Multiple Sclerosis | Arthritis, Rheumatoid | 0.999277702 |
| 15 | Guillain-Barre Syndrome | Anemia, Hemolytic, Autoimmune | 0.999261647 |
| 16 | Pemphigoid, Bullous | Myasthenia Gravis | 0.999164004 |
| 17 | Polyendocrinopathies, Autoimmune | Diffuse Cerebral Sclerosis of Schilder | 0.999125009 |
| 18 | Thyroiditis, Autoimmune | Anemia, Hemolytic, Autoimmune | 0.99912205 |
| 19 | Glomerulonephritis, IGA | Arthritis, Rheumatoid | 0.999070586 |
| 20 | Thyroiditis, Autoimmune | Pemphigoid, Bullous | 0.99901786 |
| 21 | Still's Disease, Adult-Onset | Purpura, Thrombocytopenic, Idiopathic | 0.999014866 |
| 22 | Glomerulonephritis, IGA | Addison Disease | 0.999014768 |
| 23 | Pemphigoid, Bullous | Guillain-Barre Syndrome | 0.99901189 |
| 24 | Lambert-Eaton Myasthenic Syndrome | Dermatitis Herpetiformis | 0.998904859 |
| 25 | Graves Disease | Addison Disease | 0.998886601 |
| 26 | Sjogren's Syndrome | Dermatitis Herpetiformis | 0.998833748 |
| 27 | Glomerulonephritis, IGA | Giant Cell Arteritis | 0.998721668 |
| 28 | Hepatitis, Autoimmune | Diabetes Mellitus, Type 1 | 0.998692806 |
| 29 | Thyroiditis, Autoimmune | Purpura, Thrombocytopenic, Idiopathic | 0.998665224 |
| 30 | Multiple Sclerosis | Hepatitis, Autoimmune | 0.998629445 |
| 31 | Purpura, Thrombocytopenic, Idiopathic | Myasthenia Gravis | 0.998621968 |
| 32 | Sjogren's Syndrome | Hepatitis, Autoimmune | 0.99851194 |
| 33 | Myasthenia Gravis | Anemia, Hemolytic, Autoimmune | 0.998497758 |
| 34 | Thyroiditis, Autoimmune | Guillain-Barre Syndrome | 0.998497668 |
| 35 | Still's Disease, Adult-Onset | Pemphigoid, Bullous | 0.998482826 |
| 36 | Lambert-Eaton Myasthenic Syndrome | Diabetes Mellitus, Type 1 | 0.998469513 |
| 37 | Graves Disease | Giant Cell Arteritis | 0.998432214 |
| 38 | Still's Disease, Adult-Onset | Graves Disease | 0.998244814 |
| 39 | Lupus Erythematosus, Systemic | Glomerulonephritis, IGA | 0.998215699 |
| 40 | Sjogren's Syndrome | Diabetes Mellitus, Type 1 | 0.998169826 |
| 41 | Multiple Sclerosis | Lambert-Eaton Myasthenic Syndrome | 0.998155645 |
| 42 | Myasthenia Gravis | Guillain-Barre Syndrome | 0.998065377 |
| 43 | Sjogren's Syndrome | Arthritis, Rheumatoid | 0.998010498 |
| 44 | Lupus Erythematosus, Systemic | Hepatitis, Autoimmune | 0.997963322 |
| 45 | Pemphigoid, Bullous | Anemia, Hemolytic, Autoimmune | 0.997936699 |
| 46 | Uveomeningoencephalitic Syndrome | Diffuse Cerebral Sclerosis of Schilder | 0.99789763 |
| 47 | Hepatitis, Autoimmune | Dermatitis Herpetiformis | 0.997736878 |
| 48 | Lupus Erythematosus, Systemic | Lambert-Eaton Myasthenic Syndrome | 0.997705848 |
| 49 | Sjogren's Syndrome | Multiple Sclerosis | 0.997605224 |
| 50 | Sjogren's Syndrome | Lupus Erythematosus, Systemic | 0.997572116 |
