# Supplementary material for: Calculation of Similarity Between 26 Autoimmune Diseases Based on Three Measurements Including Network, Function, and Semantics
Source: Front Genet. 2021 Nov 11;12:758041. doi: 10.3389/fgene.2021.758041 (PMC8632457; doi:10.3389/fgene.2021.758041)
Supplement: Supplementary file 3 [file Table3.DOCX]

**Supplementary Table 3** Top 50 pairs of autoimmune diseases ranked by functional similarity scores.

| **Rank** | **Autoimmune disease** | **Autoimmune disease** | **FunSim score** |
| --- | --- | --- | --- |
| 1 | Uveomeningoencephalitic Syndrome | Polyendocrinopathies, Autoimmune | 0.873933251 |
| 2 | Graves Disease | Thyroiditis, Autoimmune | 0.722867689 |
| 3 | Addison Disease | Hepatitis, Autoimmune | 0.663736502 |
| 4 | Myasthenia Gravis | Thyroiditis, Autoimmune | 0.654207508 |
| 5 | Uveomeningoencephalitic Syndrome | Addison Disease | 0.625890827 |
| 6 | Thyroiditis, Autoimmune | Hepatitis, Autoimmune | 0.625387301 |
| 7 | Arthritis, Rheumatoid | Lupus Erythematosus, Systemic | 0.623997482 |
| 8 | Myasthenia Gravis | Hepatitis, Autoimmune | 0.606878785 |
| 9 | Polyendocrinopathies, Autoimmune | Pemphigoid, Bullous | 0.582925048 |
| 10 | Addison Disease | Thyroiditis, Autoimmune | 0.578253279 |
| 11 | Myasthenia Gravis | Addison Disease | 0.540623155 |
| 12 | Uveomeningoencephalitic Syndrome | Pemphigoid, Bullous | 0.540273289 |
| 13 | Pemphigus | Uveomeningoencephalitic Syndrome | 0.525140786 |
| 14 | Anemia, Hemolytic, Autoimmune | Purpura, Thrombocytopenic, Idiopathic | 0.5 |
| 15 | Still's Disease, Adult-Onset | Guillain-Barre Syndrome | 0.5 |
| 16 | Myasthenia Gravis | Uveomeningoencephalitic Syndrome | 0.5 |
| 17 | Lupus Erythematosus, Systemic | Multiple Sclerosis | 0.494141232 |
| 18 | Sjogren's Syndrome | Thyroiditis, Autoimmune | 0.487688038 |
| 19 | Sjogren's Syndrome | Addison Disease | 0.487647481 |
| 20 | Myasthenia Gravis | Graves Disease | 0.486233719 |
| 21 | Polyendocrinopathies, Autoimmune | Addison Disease | 0.485518473 |
| 22 | Uveomeningoencephalitic Syndrome | Hepatitis, Autoimmune | 0.485518473 |
| 23 | Graves Disease | Multiple Sclerosis | 0.480224541 |
| 24 | Myasthenia Gravis | Sjogren's Syndrome | 0.474152278 |
| 25 | Arthritis, Rheumatoid | Multiple Sclerosis | 0.466355045 |
| 26 | Sjogren's Syndrome | Graves Disease | 0.464430225 |
| 27 | Sjogren's Syndrome | Hepatitis, Autoimmune | 0.463815966 |
| 28 | Graves Disease | Hepatitis, Autoimmune | 0.446173053 |
| 29 | Diabetes Mellitus, Type 1 | Arthritis, Rheumatoid | 0.445015612 |
| 30 | Diabetes Mellitus, Type 1 | Lupus Erythematosus, Systemic | 0.439946708 |
| 31 | Multiple Sclerosis | Thyroiditis, Autoimmune | 0.433001116 |
| 32 | Diabetes Mellitus, Type 1 | Multiple Sclerosis | 0.42645979 |
| 33 | Sjogren's Syndrome | Multiple Sclerosis | 0.425714203 |
| 34 | Uveomeningoencephalitic Syndrome | Thyroiditis, Autoimmune | 0.423773388 |
| 35 | Pemphigus | Sjogren's Syndrome | 0.420702444 |
| 36 | Arthritis, Rheumatoid | Graves Disease | 0.418571568 |
| 37 | Glomerulonephritis, IGA | Graves Disease | 0.417442018 |
| 38 | Graves Disease | Addison Disease | 0.416366389 |
| 39 | Guillain-Barre Syndrome | Giant Cell Arteritis | 0.413334647 |
| 40 | Sjogren's Syndrome | Uveomeningoencephalitic Syndrome | 0.409217497 |
| 41 | Myasthenia Gravis | Giant Cell Arteritis | 0.403750893 |
| 42 | Guillain-Barre Syndrome | Purpura, Thrombocytopenic, Idiopathic | 0.4 |
| 43 | Still's Disease, Adult-Onset | Purpura, Thrombocytopenic, Idiopathic | 0.4 |
| 44 | Diabetes Mellitus, Type 1 | Graves Disease | 0.397928878 |
| 45 | Myasthenia Gravis | Polyendocrinopathies, Autoimmune | 0.397242387 |
| 46 | Pemphigus | Addison Disease | 0.397041787 |
| 47 | Pemphigus | Hepatitis, Autoimmune | 0.390914795 |
| 48 | Sjogren's Syndrome | Giant Cell Arteritis | 0.385048331 |
| 49 | Giant Cell Arteritis | Purpura, Thrombocytopenic, Idiopathic | 0.382874647 |
| 50 | Still's Disease, Adult-Onset | Giant Cell Arteritis | 0.376906561 |
