# Supplementary material for: Calculation of Similarity Between 26 Autoimmune Diseases Based on Three Measurements Including Network, Function, and Semantics
Source: Front Genet. 2021 Nov 11;12:758041. doi: 10.3389/fgene.2021.758041 (PMC8632457; doi:10.3389/fgene.2021.758041)
Supplement: Supplementary file 4 [file Table4.DOCX]

**Supplementary Table 4** Top 50 pairs of autoimmune diseases ranked by semantic similarity scores.

| Rank | Autoimmune disease | Autoimmune disease | SemSim score |
| --- | --- | --- | --- |
| 1 | Uveomeningoencephalitic Syndrome | Polyendocrinopathies, Autoimmune | 0.4 |
| 2 | Graves Disease | Thyroiditis, Autoimmune | 0.307692308 |
| 3 | Anemia, Hemolytic, Autoimmune | Purpura, Thrombocytopenic, Idiopathic | 0.25 |
| 4 | Uveomeningoencephalitic Syndrome | Addison Disease | 0.25 |
| 5 | Still's Disease, Adult-Onset | Guillain-Barre Syndrome | 0.25 |
| 6 | Polyendocrinopathies, Autoimmune | Pemphigoid, Bullous | 0.25 |
| 7 | Myasthenia Gravis | Thyroiditis, Autoimmune | 0.24137931 |
| 8 | Arthritis, Rheumatoid | Lupus Erythematosus, Systemic | 0.235294118 |
| 9 | Addison Disease | Hepatitis, Autoimmune | 0.235294118 |
| 10 | Myasthenia Gravis | Uveomeningoencephalitic Syndrome | 0.230769231 |
| 11 | Myasthenia Gravis | Hepatitis, Autoimmune | 0.222222222 |
| 12 | Pemphigus | Uveomeningoencephalitic Syndrome | 0.222222222 |
| 13 | Myasthenia Gravis | Addison Disease | 0.210526316 |
| 14 | Guillain-Barre Syndrome | Purpura, Thrombocytopenic, Idiopathic | 0.2 |
| 15 | Still's Disease, Adult-Onset | Purpura, Thrombocytopenic, Idiopathic | 0.2 |
| 16 | Guillain-Barre Syndrome | Giant Cell Arteritis | 0.2 |
| 17 | Uveomeningoencephalitic Syndrome | Pemphigoid, Bullous | 0.2 |
| 18 | Polyendocrinopathies, Autoimmune | Addison Disease | 0.181818182 |
| 19 | Uveomeningoencephalitic Syndrome | Hepatitis, Autoimmune | 0.181818182 |
| 20 | Addison Disease | Thyroiditis, Autoimmune | 0.178571429 |
| 21 | Sjogren's Syndrome | Uveomeningoencephalitic Syndrome | 0.176470588 |
| 22 | Sjogren's Syndrome | Addison Disease | 0.173913043 |
| 23 | Myasthenia Gravis | Sjogren's Syndrome | 0.166666667 |
| 24 | Graves Disease | Addison Disease | 0.166666667 |
| 25 | Myasthenia Gravis | Polyendocrinopathies, Autoimmune | 0.166666667 |
| 26 | Graves Disease | Multiple Sclerosis | 0.166666667 |
| 27 | Myasthenia Gravis | Graves Disease | 0.162790698 |
| 28 | Arthritis, Rheumatoid | Multiple Sclerosis | 0.156028369 |
| 29 | Lupus Erythematosus, Systemic | Multiple Sclerosis | 0.152671756 |
| 30 | Pemphigus | Sjogren's Syndrome | 0.15 |
| 31 | Thyroiditis, Autoimmune | Hepatitis, Autoimmune | 0.148148148 |
| 32 | Graves Disease | Hepatitis, Autoimmune | 0.146341463 |
| 33 | Pemphigus | Hepatitis, Autoimmune | 0.142857143 |
| 34 | Guillain-Barre Syndrome | Antiphospholipid Syndrome | 0.142857143 |
| 35 | Pemphigus | Lambert-Eaton Myasthenic Syndrome | 0.142857143 |
| 36 | Sjogren's Syndrome | Multiple Sclerosis | 0.138461538 |
| 37 | Diabetes Mellitus, Type 1 | Arthritis, Rheumatoid | 0.136585366 |
| 38 | Sjogren's Syndrome | Hepatitis, Autoimmune | 0.136363636 |
| 39 | Sjogren's Syndrome | Giant Cell Arteritis | 0.136363636 |
| 40 | Uveomeningoencephalitic Syndrome | Thyroiditis, Autoimmune | 0.136363636 |
| 41 | Diabetes Mellitus, Type 1 | Graves Disease | 0.135135135 |
| 42 | Pemphigus | Addison Disease | 0.133333333 |
| 43 | Arthritis, Rheumatoid | Graves Disease | 0.130081301 |
| 44 | Multiple Sclerosis | Thyroiditis, Autoimmune | 0.128571429 |
| 45 | Sjogren's Syndrome | Graves Disease | 0.127659574 |
| 46 | Pemphigus | Polyendocrinopathies, Autoimmune | 0.125 |
| 47 | Myasthenia Gravis | Pemphigus | 0.125 |
| 48 | Sjogren's Syndrome | Polyendocrinopathies, Autoimmune | 0.125 |
| 49 | Sjogren's Syndrome | Thyroiditis, Autoimmune | 0.121212121 |
| 50 | Pemphigus | Thyroiditis, Autoimmune | 0.12 |
